# Human Umbilical Cord Blood Stem Cells: Rational for Use as a Neuroprotectant in Ischemic Brain Disease

**DOI:** 10.3390/ijms11093513

**Published:** 2010-09-21

**Authors:** Hadar Arien-Zakay, Shimon Lecht, Arnon Nagler, Philip Lazarovici

**Affiliations:** 1 The School of Pharmacy Institute for Drug Research, Faculty of Medicine, The Hebrew University of Jerusalem, Jerusalem, Israel; E-Mails: hadara@ekmd.huji.ac.il (H.A.-Z.); shimonl@ekmd.huji.ac.il (S.L.); 2 Division of Hematology and Cord Blood Bank, Chaim Sheba Medical Center, Tel-Hashomer, Israel; E-Mail: arnon.nagler@sheba.health.gov.il (A.N.)

**Keywords:** human umbilical cord blood stem cells, brain ischemia, neuroprotection

## Abstract

The use of stem cells for reparative medicine was first proposed more than three decades ago. Hematopoietic stem cells from bone marrow, peripheral blood and human umbilical cord blood (CB) have gained major use for treatment of hematological indications. CB, however, is also a source of cells capable of differentiating into various non-hematopoietic cell types, including neural cells. Several animal model reports have shown that CB cells may be used for treatment of neurological injuries. This review summarizes the information available on the origin of CB-derived neuronal cells and the mechanisms proposed to explain their action. The potential use of stem/progenitor cells for treatment of ischemic brain injuries is discussed. Issues that remain to be resolved at the present stage of preclinical trials are addressed.

## 1. Introduction

Ischemic brain and spinal cord injuries, as well as neurodegenerative diseases, represent poorly managed diseases that are major targets for pharmacological intervention. Modern approaches are based on cell and gene therapy [1]. They include implantation/transplantation of neuronal cells or cells engineered with specific gene encodings for neuroprotective growth factors. For example, human fetal mesencephalic dopaminergic tissue and chromaffin cells when transplanted into damaged tissues of the central nervous system (CNS) were found to replace lost dopaminergic phenotype [2,3]. However, clearly there are ethical concerns and a limit to the supply of the tissues mentioned as well as recent concerns with side effects [3]. Alternative sources of tissue have been investigated, and stem cells are an attractive renewable tissue supply. Although not yet clinically available for central nervous system disorders, stem cell technology is expected to evolve into one of the most powerful tools in the biological management of complex central nervous system disorders, many of which currently have limited treatment modalities.

Neural stem cells are multipotent precursors that both self-renew and give rise to neuronal and glial progenitors [4]. They are present in the developing [5] and also in the adult central nervous system of mammals, including humans [6,7]. Neural stem cells were isolated from embryonic, bone marrow and adult subsets of both human and murine origin, and in the last few years from human umbilical cord blood (CB) [8,9]. Some sub-populations isolated from CB have been shown to differentiate into neural-like cells and when administered in animal models of brain ischemia, neurodegenerative diseases and spinal cord injuries they exhibited therapeutic effect [8,9]. Recent advances in understanding the unique biology of CB will further expand indications for its use in different settings, including those beyond transplantation for hematopoietic-related illnesses, such as neurological-related diseases [10,11].

## 2. CB—An Attractive Potential Source for Brain Regeneration

Considering its growing use for hematological reconstitution, its widespread availability and the potential use in non-hematopoietic related-diseases, CB is an attractive source for tissue regeneration [12,13]. With the annual global birth rate of over 100 million per year and the fast availability of cryopreserved units, CB is a large underutilized stem cell source with many innate advantages. For the treatment of brain injuries, the collection of stem cells from CB of a placental umbilical cord stump is simpler compared to the more complex collection from bone marrow or from embryonic human brain. Furthermore, a paramount advantage of CB stem cells over adult stem cells is that they possess a primitive ontogeny and have not been exposed to immunologic challenge. This rather naïve immune system of CB cells may play a significant role in reduced rejection after their transplantation into a mismatched host [12]. Indeed, when comparing the records of recipients of CB from human leukocyte antigen (HLA)-matched siblings and recipients of HLA-matched bone marrow, a lower risk of acute graft-versus-host-disease (GVHD) and chronic GVHD among the CB transplant patients was found [12,14]. Another advantage of CB is the increasing number of public banks and the eligibility of its use for transplantations. While research with embryonic stem cells continues to generate considerable controversy, human umbilical stem cells provide an alternative cell source that has been more ethically acceptable and appears to have widespread public support, with 43 public CB banks in 26 countries [15].

The successful transplantations of CB as an alternative to bone marrow for the treatment of hematopoietic-related diseases [12] provide important evidence that CB-related therapies are feasible and may be further suggested to non-hematopoietic applications, such as brain injuries. Currently, CB is considered a second best choice after matched bone marrow. However, results of recent international studies indicate that in particular clinical settings, such as in children with leukemia, CB may become a frontline hematopoietic stem cell source for transplantation. With these advantages, however, the CB limited cell dose remains a main setback of CB transplantations, particularly in adult population. New strategies, such as transplantation with two cord blood units or using non-myeloablative conditioning, have remarkably expanded the availability of CB transplants in adults with hematological malignancies [16]. Clinical trials with *in vitro* expanded CB-derived stem cells are under way [12].

Considering its many advantages and since CB was found to contain a mixture of different types of stem cells in numbers not seen in any other location, including embryonic- like stem cells, hematopoietic stem cells, endothelial stem cells, epithelial stem cells, mesenchymal stem cells, unrestricted somatic stem cells and neuronal stem cells [8,9], we believe it may become a frontline source of cells for therapy, including for the treatment of brain injuries.

## 3. Neural Stem Cells from CB—An Unclear Origin

During the last decade, numerous *in vitro* studies have demonstrated generation of neuronal cells from CB progenitors [8,9]. The experimental approaches to induce neural differentiation were based on supplementation of growth factors such as NGF [17] and interferon-gamma [18] as well as of chemical agents, such as retinoic acid [19–22], dimethylsulfoxide [23] and beta-mercaptoethanol [24]. The induction of neuronal phenotypes was characterized by the expression of typical neuronal markers specific to different stages of neural development and by functional proteins, including voltage- and ligand-gated ionic channels [25]. For instance, McGuckin *et al*. demonstrated the presence of embryonic stem (ES)-like cells in CB, from which under expansion with thrombopoietin, flt-3, and c-kit ligand, slow-dividing adherent cell populations resulted with neuroglial progenitor morphology, upregulation of primitive neuroglial cell markers, and expression of glial fibrillary acidic protein (GFAP) [26]. Our group isolated a subpopulation of collagen-adherent cells from CB [17]. Under *in vitro* treatment with neuronal conditioning medium, NGF and interferon-gamma, these cells differentiated into a neuronal-like lineage, expressing neuronal (NeuN, neuron specific enolase-NSE, neurofilaments, microtubulin associated protein-MAP-2) and glial cells (GFAP) markers [18]. Chen *et al*. also characterized a subpopulation of adherent cells in the CB, expressing vimentin, nestin, and A2B5, antigens typically found in brain tissue, and the neurotrophic receptors, trk-A, trk-B and trk-C, suggesting the cells ability to differentiate by growth factors towards a neural phenotype [27]. Buzanska *et al*. established a clonogenic non-immortalized CB neural stem cell line that could be maintained in culture at different stages of neural progenitor development with trophic factors, mitogens and neuromorphogens [28]. In parallel, Rogers *et al*. have isolated a population of CD45+ cells from CB with multipotential properties [29]. These stem cells were isolated by culture in a serum free, growth factor supplemented medium and were capable of differentiating into bone, muscle, neural, blood and endothelial cells after exposure to specialized differentiation media. The full range of neural differentiation ability of the cells was demonstrated by the achievement of positive phenotypic and functional indicators for dopaminergic neurons, oligodendrocytes and astrocytes [29]. Finally, electrical activity of CB-derived CD34^−^/CD45^−^ differentiating cells was recorded using whole-cell patch-clamp [25]. In these cells, gene and partially protein expression for voltage-dependent potassium and sodium channels and the neurotransmitter receptors acetylcholine (ACh), gamma-aminobutyric acid (GABA), glutamate, glycine, 5-hydroxytryptamine (5-HT), and dopamine (DA) were identified and the cells further displayed an inward rectifying potassium current (Kir) and an outward rectifying potassium current (I(K+)). Kainic acid (KA), a non-*N*-methyl-d-asparate (NMDA) glutamate-receptor agonist, induced an inward current in some of the cells, while KA, glycine, DA, ACh, GABA, and 5-HT partially blocked Kir through their respective receptors, indicating differentiation toward neuron-like cells, with functional voltage- and ligand-gated channels identified in other neuronal systems [25].

In similarity to the diversity in the reported differentiating methods, the cell type or subpopulation of cells, which is the source of neurons and glia from CB, also remains undefined. Most studies used heterogeneous or poorly characterized populations of CB cells. Some were defined according to their classification as hematopoietic and non-hematopoietic stem cells and there were no standardized criteria for determination of their origin. To date it is common to isolate and characterize hematopoietic and non-hematopoietic stem cells by the expression or lack of expression of CD34, CD45 and/or CD133 antigens, respectively.

Subpopulations of CB isolated according to the expression of hematopoietic stem cells markers such as CD34^+^ [30], CD133^+^ [21,31] or CD45^+^ [29] were induced *in vitro* to differentiate towards neuronal-like phenotype. Subsequently, CD34^−^ CD45^−^ non-hematopoietic stem cells [20,22,25], and MSC [23,32] and unrestricted somatic stem cells (USSCs) [33,34] were identified as origins of the neuronal-like cells. Classically, MSC are defined as being able to adhere to plastic, expressing CD29, CD73, CD44, CD90, CD105 antigens, and not expressing the hematopoietic cell markers CD34, CD45, and MHC class II antigen [35], although none of these markers appears to be exclusively expressed on MSC [36]. The MSC origin was determined by the expression of various MSC markers and was further supported by showing that some CB-derived progenitors adhere to plastic and poly-l-lysine [19,27,37,38]. We isolated a subpopulation of cells, capable of differentiating into a neuronal phenotype using their collagen adherent properties and positive expression of alpha1 and alpha2 collagen-receptors [17]. The microarray gene expression analysis of these cells indicates that the cells are negative for the hematopoietic markers CD34, CD49c, CD49d, CD62e, CD62p, CD106, CD117, CD133, CD235a, HLA-DRB4, HLA-DRB4 and HAS1, and positive for the mesenchymal markers CD13, CD29, CD44, CD49a/b, CD49e, CD73, CD105 and vimentin [18], supporting their USSCs/MSC origin.

Altogether, the uncertainty in the precise origin of the CB-cells differentiating into a neuronal-like phenotype (hematopoietic or non-hematopoietic progenitors), is reflecting on our inability to determine whether a primitive multipotent stem cell resides in CB or whether a transdifferentiation process [39] is responsible for neuronal differentiation from hematopoietic lineage. The transdifferentiation hypothesis, proposed during the last decade, is challenging the concept that a cell committed to a specific phenotypic fate, by virtue of residence in a mature organ, cannot change its destiny. However, one must be cautious when interpreting transdifferentiation whereas the molecular mechanisms responsible for stem cell plasticity are not completely understood. It is also possible that the stem cells found in adult tissues are true multipotent stem cells that arrive in the adult tissue early in development (or perhaps migrate to the organ later in development), but retain “stemness” (self-renewal, multipotency) in the adult tissue throughout life [39].

In this view, the specific identification of the CB-derived cells with neural differentiation abilities, as hematopoietic or non-hematopoietic source may be crucial for their further definition as neuronal or non-neuronal originated stem cells. However, even if originated from a non-neuronal stem cell, once have the ability to differentiate into a neuronal phenotype and provide neuroprotection against neurological deficits, CB cell populations are of high clinical relevance.

## 4. Therapeutic Effect by CB-Derived Cells on Ischemic Brain Injury

Cerebral ischemia induces death of all neural cell types within the region affected by the loss of blood flow. On the other hand, neurodegenerative diseases, such as Amyotrophic lateral sclerosis, Alzheimer’s, Huntington’s and Parkinson’s diseases involve degeneration of defined neuronal phenotypes in the CNS [8]. In both cases, most therapies for these diseases are palliative rather than restorative and the quality of life of the affected individuals is greatly impaired. Stem cells may confer neuroprotection which holds the promise of resulting in the replacement or regeneration of damaged neurological tissues. "Neuroprotection" was coined to describe interventions protecting the brain from pathological damage. The neuroprotective drug/approach aims at preventing the death of cells by inhibiting insult-activated pathological step(s) and/or induction of biochemical pathways that induce survival. This drug/approach may also confer neuroprotection by induction of neurogenesis, as observed by proliferation and migration of endogenous stem cells to the site of brain injury, or upon exogenous transplantation [40]. It was shown that in stroke, neuroprotection involves the inhibition of pathological events leading to calcium influx, activation of free radical reactions and cell death [41]. It is important to note, however, that the cell therapy strategy may differ according to the pathophysiology of the disease. Whereas in chronic neurodegenerative diseases a defined neuronal pathway is degenerated, in acute ischemic brain injuries the damage occurs within various regions of the brain. A rational treatment for ischemic brain injuries will therefore include the support of the injured cell survival and an induction of endogenous neurogenesis, while for the treatment of neurodegenerative diseases a cell replacement approach may be more effective.

A major challenge is to develop a neuroprotective therapy for ischemic brain patients that can be applied in the early stages of the insult to slow, stop, or reverse the progression of the disease. Over the last two decades, more than 500 drugs demonstrated neuroprotective properties in pre-clinical and clinical setups, however, none have reached the stage of an “approved” therapy [42,43]. Innovative approaches, such as neuroprotection by stem cells, raise hope in the clinic. Indeed, recent animal studies and preclinical trials have provided evidence that cell-based, regenerative therapies can lead to functional recovery of brain ischemia patients. Stem cells can differentiate into neuronal or neuron-like cells that may replace lost neurons or provide trophic support to tissue at risk in the infarcted area of the brain and help to promote survival and neuroprotection [44]. However, stem cell–based therapy is problematic because aborted human fetuses from which human neuronal stem cells are isolated are scarce and raise complicated ethical issues. High-quality, neuronal progenitor cells from CB, if available in sufficient quantity, might provide a viable alternative.

The ability of CB and derived cell populations to award protection against neurological deficits was shown *in vivo* in models of ischemic brain injuries. Using a transient (two-hour) MCAO (medial carotid artery occlusion) model of stroke, Chen *et al.* were the first to demonstrate that upon CB intravenous administration, many of the physical and behavioral deficits were ameliorated [45]. A significant improvement in functional outcome on motor and modified neurological severity score (mNSS) tests was found in animals given CB cells at 1 day after stroke. At 14 and 35 days after transplantation, intravenously injected CB cells were found in the brain, and significantly more CB cells were found in the ipsilateral hemisphere than in the contralateral hemisphere. Many cells migrated into the boundary zone of ischemic brain. CB cells survive, and some express cell type– specific neuronal markers NeuN (2%) and MAP-2 (3%), astrocye marker GFAP (6%) and endothelial cell marker FVIII (8%). *In vitro*, using a brain tissue extract assay, a significant CB cells migration activity was shown in the presence of ischemic cerebral tissue harvested at 24 hours after MCAO compared with normal nonischemic brain tissue [45]. Furthermore, using ischemic brain extracts, Newman *et al*. suggested that the migration of CB-cells into the ischemic area is triggered by cytokines and chemokines released in the ischemic tissue [46]. These observations were further supported by other groups, monitoring the dependence of the beneficial effects on the CB cell dose [47], mode of cell implantation [48] and the timing of transplantation after injury [49]. Using a permanent MCAO, Vendrame *et al*. found an inverse relationship between CB cells dose and damaged infarct volume [47]. Furthermore, at four weeks after intravenous infusion, there was a significant recovery in behavioral performance only when 10^6^ or more CB cells were delivered [47]. Intravenous delivery was suggested to be more effective than striatal delivery in producing long-term (two-months after implantation) functional benefits to the stroked animals [48]. The therapeutic efficacy of the treatment was demonstrated even when cells were administrated 48 hours after the injury [49]. Successful treatment at this time point should offer encouragement to clinicians that a therapy with a broader window of efficacy may be available to treat ischemic stroke.

*In vivo* animal studies succeeded in using CB also for the treatment of heatstroke. Under an exposure to an ambient temperature of 43 degrees C, rats transplanted with intravenous or intracerebroventricular CB cells showed a significant improvement in their survival as composed to the non-transplanted group (61–148 min. *vs*. 21–23 min., respectively) [50]. The circulatory shock, intracranial hypertension, cerebral hypoperfusion and hypoxia, increment of cerebral ischemia, and damage markers during heat stroke were all significantly attenuated by the delivery of CB-cells but not peripheral blood mononuclear cells. Furthermore, treatment with CB-derived CD34+ cells significantly improved survival time (63–291 min) while causing attenuation of hypotension, hepatic and renal failure, hypercoagulable state, activated inflammation, and cerebral ischemia and injury heatstroke reactions. In addition, the levels of IL-10 in plasma and glial cell line-derived neurotrophic factors in brain were all significantly increased after CB-CD34+ cell therapy during heatstroke [51]. This data indicate that CB, but not peripheral blood, cell therapy may resuscitate persons who had a heatstroke by reducing multiorgan dysfunction or failure.

Traumatic brain injury is also a potential target for treatment using CB cells as was first indicated by Lu *et al*. [52]. They documented migration of intravenous transplanted cells into the parenchyma of brain lesion and a decrease in neurological damage in a rat model. The cells expressed the neuronal markers, NeuN and MAP-2, and the astrocytic marker, GFAP. Some CB cells integrated into the vascular walls within the boundary zone of the injured area [52].

Treatment with CB cells was also proposed for other brain damages, such as hypoxic-ischemic damage around birth. Using brain damaged neonatal rats, Meier *et al*. observed both incorporation of CB mononuclear cells in the lesioned brain area and an alleviation of the neurological effects of cerebral palsy as assessed by footprint and walking pattern analysis [53]. This was also observed under intracerebral transplantation of MSCs, which were found to differentiate into astrocytes, but not neurons [54]. Furthermore, a pilot study is in progress at Duke University to test the feasibility (of collection, preparation and infusion) using autologous CB on a baby born with signs of brain injury during the first 14 days after birth (http://www.clinicaltrials.gov/ct2/show/NCT00593242?order=1).

## 5. CB-Induced Therapeutic Effect on Ischemic Brain Injury—Routes for Mediating Mechanisms

Although functional improvement and reductions in lesion volume were observed in ischemic rodents treated with CB cells, cells expressing the human nuclei marker within the brain were rather scant, suggesting that the restorative effects of CB may be mediated by mechanisms other than cell replacement [55]. Some of the mechanisms proposed for these studies involves reduced inflammation [49,56,57], nerve fiber reorganization by trophic actions [55], increased cell survival and enhanced angiogenesis [58,59]. Evidences for reduced inflammation included the suppression of lymphocytes [57], granulocyte and monocyte [49] infiltration and the lack of astrocytic and microglial activation in the parenchyma [49]. They also included the rescue of spleen weight and splenic CD8+ T-cell counts [56] and an increase in the production of IL-10 while decreasing IFN-gamma [56]. To determine whether CB-cells could exert trophic effects on the host brain, Xiao *et al*. directly transplanted the cells into the brain parenchyma after ischemic brain injury and showed an increased sprouting of nerve fibers from the non damaged hemisphere into the ischemically damaged side of the brain [55]. Their results suggest that restorative effects observed with CB-cells treatment following ischemic brain injury may be mediated by trophic actions that result in the reorganization of host nerve fiber connections within the injured brain. CB-MCSs [58] and CB-CD34 positive cells [59] were suggested to promote either directly or indirectly an environment conducive to neovascularization of ischemic brain so that neuronal regeneration can proceed. Ding *et al*. showed that transplanted CB-MSCs migrated towards the ischemic boundary zone, were able to differentiate into glial, neuronal, doublecortin+, CXCR4+, and vascular endothelial cells and that CB-cells transplantation promoted the formation of new blood vessels [58]. These results suggest a potential enhanced neuroplasticity as well as an increase local cortical blood flow in the ischemic hemisphere, supporting brain plasticity and recovery. Furthermore, in a canine thromboembolic brain ischemia model, transplanted CB-MSCs had differentiated into neurons and astrocytes and were observed in and around endothelial cells that were positive for von Willebrand factor (vWF) [60]. These cells expressed neuroprotective factors, such as brain-derived neurotrophic factor (BDNF) and vascular endothelial growth factor (VEGF), at four weeks after the transplantation, in correlation to reduced infarct volume and earlier recovery from the neurological deficit [60].

We and others have widely explored these mechanisms *in vitro* [61–63]. CB-derived progenitors were shown to confer neuroprotection in models of cerebral ischemia using oxygen glucose deprivation (OGD) insulted adrenal medulla neurons [61], primary rat cortical neuronal cultures exposed to hypoxia and hippocampal slice cultures exposed to OGD [57,62], as well as on differentiated neuroblastoma SH-SY5Y cells exposed to hypoxia [63]. This protection is rationalized by a mechanism involving the release of antioxidants, the decrease in numbers of free radicals in the injured neuron and the accumulation of growth factors in the media. This includes the release of the nerve growth factor (NGF), VEGF and basic fibroblast growth factor (bFGF) and the modulations of neurotrophic and angiogenic factors gene expression [61]. As these CB cells selectively home into the lesioned brain areas [45,46,52,53,58], the release of neurotrophic and angiogenic factors [64,65] and/or antioxidants *in vivo*, as shown *in vitro*, would be highly specific and locally limited to the damaged brain area, supporting a ”bystander” neuroprotective strategy [66].

A “bystander” protection of the ischemic neuron refers to an effect mediated by soluble growth factors and not by neurogenesis (proliferation and differentiation of neuronal stem cells replacing neuronal network). The concept of bystander cell therapy is expected to replace or complement the common method of providing neurotrophins to the injured brain [67]. Clinical trials testing the therapeutic properties of various neurotrophic factors have been rather disappointing [68,69]. Neurotrophins are relatively large proteins, that do not penetrate the blood-brain barrier [70] and the poor outcome of clinical trials can be partially attributed to the inability of neurotrophins to effectively reach the brain. An alternative to the administration of neurotrophins or their agonists is to use small blood-brain-barrier-penetrating molecules, which stimulate the production and release of neurotrophins in the brain [67] or, as presently suggested, to implant/transplant neuronal stem cells with the ability to secrete neurotrophins in the insulted area.

## 6. Therapeutic Effect of CB-Derived Cells on Ischemic Brain Injury—A Glance to the Future

CB-cells hold tremendous potential for the treatment of neurological diseases and are already used in the clinic for the treatment of hematological-related diseases. However, in order to be ethically accepted for the therapy of neurological disorders many issues should be resolved with pre-clinical studies before proceeding with clinical treatment.

An important question is which of the various CB population(s) is the one that best induces neuroprotection. This question must be separately addressed for each neurological disease, since the leading pathophysiologal processes are different in chronic neurodegenerative diseases from those in acute brain ischemia. The answer will determine which cell-population(s) to use for treatment and the strategy for cell therapy. Whereas for the treatment of ischemic brain injuries neuroprotection by a “bystander” effect may be more suitable for the coincidence nature of these diseases, the treatment of degenerated defined neuronal phenotype in neurodegenerative diseases may involve both cell replacement and neuroprotection strategies. Furthermore, even though transplantation of whole CB may be simpler, it contains several types of cells and may either induce a stronger immune response and/or may be less effective. The risk of graft versus host disease (GvHD) in CB transplantations for neurological applications has to be also quantified. In spite of the naïve nature of the immune system of a newborn and reports of lower incidence of GvHD for hematological applications [71], this complication is still viable and need to be addressed. Other issues concerning the use of CB for hematological-related diseases should also be considered regarding its potential use as a neuroprotectant in ischemic brain injuries. One prime concern is the limited cell dose remains a main setback of CB transplantations, particularly in the adult population. Currently, a single unit of CB only provides cells in sufficient quantity to treat successfully neonatal or small-stature pediatric patients with hematological malignancies and other conditions requiring lymphohematopoietic reconstitution. Whether a single CB unit will be sufficient for therapy of ischemic brain is to be determined. Given ischemic brain injuries generally occurs in adult patient, most probably their treatment with CB will include new strategies such as transplantation with two cord blood units or using non-myeloablative conditioning, which have remarkably expanded the availability of CB transplants in adults with hematological malignancies [12,16]. *In vitro* expansion of CB-derived cells before transplantation is also a potential possibility. Finally, considering the currently un-uniformed protocols for CB-cells isolation and transplantation in ischemic brain injuries animal models, it is difficult to evaluate if there is a significant impact of biological differences intrinsic to individual units of CB on the reproducibility of results observed. This issue is to be addressed in a coherent research in order to predict the expectations in human subjects.

A better understanding of the mechanism and identification of specific cell populations will provide stronger rationale for considering initiation of clinical investigations in humans. One of the mechanisms to be solved is the identity of the released factors after transplantation. For example, cytokines and chemokines enhance the expression of adhesion molecules on cerebral endothelial cells, resulting in further impairing the cerebral blood flow. Therefore, even if CB cells cannot induce neurogenesis in an injured brain, their transplantation may still result with neuroprotection due to the secretion of factors which impair the cerebral blood flow and survival of damaged brain area. Furthermore, to date there is mounting evidence that inflammation plays an important role in cerebral ischemia. Experimentally and clinically, response to ischemic injury is associated with an acute and prolonged inflammatory process characterized by the activation of resident glial cells, production of inflammatory cytokines as well as leukocyte and monocyte infiltration in the brain [72]. These are all events that may contribute to ischemic brain injury and affect recovery and plasticity of the brain [72]. However, whether the post-ischemic inflammatory response is deleterious or beneficial to the recovery of the brain, is presently a matter of debate and controversies [73]. Several investigators found a suppression of certain compartments of inflammation process in stroke animals after CB-cells transplantation [49,56,57]. However, CB-neuronal-like cells originate in a hematologic tissue and since their hematopoietic or non-hematopoietic nature is yet to be determined, an increased inflammatory effect upon their transplantation is also a realistic possibility and therefore might either be beneficial or contribute to the evolution of tissue damage.

Another main issue relates to the therapeutic time window for cell transplantation. To date, the Food and Drug Administration (FDA)-approved drug treatment for acute ischemic stroke is the anticoagulant thrombolytic tissue-plasminogen activator (t-PA), which must be administered within the first 3–4.5 h after the onset of acute ischemia [74]. Unfortunately, many of the stroke and brain trauma victims do not reach a medical facility within this narrow time window. In early reports, using bone marrow stem cells, a significant reduction in the core of the ischemic lesion was achieved at early intervention-, ranging from 3–12 h after stroke onset [75,76]. Later reports of intracerebral, intravenous or intra-arterial administration of bone marrow stem cells, starting from 24 h after transient MCAO, showed improved functional outcome in a similar experimental ischemic stroke model. Even delayed treatment seven days and even one month after the stroke onset was reported to increase brain plasticity and to improve long-term functional outcome [77,78]. Similarly, significant improvements were observed in the behavioral defects of rats that received CB cells 24–72 h after MCAO [45–49] and of traumatic brain injury [52]. The time window for effective treatment to enhance recovery from brain ischemia was found to be longer than that for acute neuroprotective stroke treatments: perhaps days or weeks, rather than minutes or hours after the ischemic onset [79].

## 7. Conclusions

CB cells have been in routine clinical practice for the past 20 years for hematological applications. The development of new therapeutic protocols in regenerative medicine requires the use of stem cells and CB is an important and readily available source of cells for these applications. CB-derived cells offer multiple advantages over adult stem cells and ES cells, including their immaturity, which may play a significant role in the rejection of generated tissue when transplanted into a mismatched host, their simple collection and their ethically acceptable for transplantations in humans.

During the last decade, a growing body of evidence suggests that CB contains cells capable of differentiating into neural phenotypes, including neurons, astroglia and oligodendroglia. A substantial diversity is currently reported regarding the identity of these cells as hematopoietic or non-hematopoietic stem cells. Furthermore, cell differentiation towards a neural phenotype involves various protocols and the multipotency properties of the isolated cells are not clear. Regarding the lack of defined handling, these CB derived-cell populations are now under investigation to determine their possible application for treatment of neurological diseases [80]. Animal models have shown that CB-cells improve recovery in neurodegenerative diseases such as amyotrophic lateral sclerosis, Parkinson’s diseases and Alzheimer’s disease [8,9]. CB-cells were also widely shown to enhance recovery in animal after ischemic brain injuries such as stroke, heatstroke, traumatic brain injury and hypoxic-ischemic damage around birth. Upon CB-cells transplantation in ischemic brain damaged animals, these cells migrated to the injured brain area and some differentiated into neuronal, astroglial and oligodendroglial phenotypes. In these transplanted animals, the damaged infarct area was decreased in a reverse correlation to the transplanted CB-cells amount, while a significant improvement in functional outcome in neurological deficits was observed. Both intravenous and striatal delivery were suggested to be effective in producing long-term functional benefits to the ischemic brain injury animals and the therapeutic efficacy of the treatment was demonstrated even when cells were administrated 48 hr after the injury. Several mechanisms of action were suggested including reduction in inflammatory response reorganization of nerve fiber by trophic actions, increase in damaged cell survival and enhancement of angiogenesis. But more is hidden than clear and comprehensive investigation of the therapeutic properties is needed to provide stronger rationale for considering initiation of clinical investigations in humans. This will include determination of the identity of the cells active in neurorecovery and the cell populations of their origin, the mechanisms of therapeutic effects, the rout of cell administration, the time window for intervention and others.

Ischemic brain injuries are known for the short time during which current available therapeutic activity is possible. However, considering the encouraging results using CB derived stem cells, their administration beyond the hyperacute phase of ischemia may amplify the intrinsic properties of the brain regarding neuroplasticity and subsequent neurological recovery. Therefore, although still in the pre-clinical stage of research, treatment by CB cells, which may extend the therapeutic window and provides significant improvements in neurological deficits, holds a tremendous potential for therapy and may create an opportunity to treat most, if not all, ischemic brain patients.
